# Anti-Listerial Activity of Bacteriocin-like Inhibitory Substance Produced by *Enterococcus lactis* LBM BT2 Using Alternative Medium with Sugarcane Molasses

**DOI:** 10.3390/antibiotics13030210

**Published:** 2024-02-23

**Authors:** Taciana Freire de Oliveira, Taís Mayumi Kuniyoshi, Elionio Galvão Frota, Sebastián Bermúdez-Puga, Letícia Naomy Sakaue, Luara Lucena Cassiano, Leonardo Tachibana, Rosane Aparecida Moniz Piccoli, Attilio Converti, Ricardo Pinheiro de Souza Oliveira

**Affiliations:** 1 Laboratory of Microbial Biomolecules, School of Pharmaceutical Sciences, University of São Paulo, Rua do Lago, 250, São Paulo 05508-000, Brazil; taciana.oliveira@usp.br (T.F.d.O.); t.kuniyoshi@alumni.usp.br (T.M.K.); egalvaofrota@gmail.com (E.G.F.); sebastianbermudez@usp.br (S.B.-P.); let.naomy@usp.br (L.N.S.); 2 Aquaculture Research Center, Scientific Research of Fisheries Institute, APTA, SAA, Av. Conselheiro Rodrigues Alves, 1252, São Paulo 04014-002, Brazil; lua.cassianolc@gmail.com (L.L.C.); ltachibana@sp.gov.br (L.T.); 3 Bionanomanufacturing Nucleus, Institute for Technological Research (IPT), Av. Prof. Almeida Prado, 532, São Paulo 05508-901, Brazil; rpiccoli@ipt.br; 4 Department of Civil, Chemical and Environmental Engineering, Pole of Chemical Engineering, University of Genoa, Via Opera Pia 15, 16145 Genoa, Italy; converti@unige.it

**Keywords:** bacteriocin-like inhibitory substance, *Enterococcus lactis*, *Listeria monocytogenes*, anti-listerial activity, sugarcane molasses

## Abstract

*Listeria monocytogenes* is a foodborne pathogen that contaminates food-processing environments and persists within biofilms on equipment, thus reaching final products by cross-contamination. With the growing demand for clean-label products, the search for natural antimicrobials as biopreservants, such as bacteriocins, has shown promising potential. In this context, this study aimed to evaluate the anti-listerial action of bacteriocins produced by *Enterococcus lactis* LBM BT2 in an alternative medium containing sugarcane molasses (SCM). Molecular analyses were carried out to characterize the strain, including the presence of bacteriocin-related genes. In the kinetic study on SCM medium *E. lactis,* LBM BT2 showed biomass and bacteriocin productions similar to those observed on a sucrose-based medium (control), highlighting the potential of the sugarcane molasses as a low-cost substrate. Stability tests revealed that the molecule remained active in wide ranges of pH (4–10) and temperature (60–100 °C). Furthermore, the proteolytic treatment reduced the biomolecule’s antimicrobial activity, highlighting its proteinaceous nature. After primary purification by salting out and tangential flow filtration, the bacteriocin-like inhibitory substance (BLIS) showed bacteriostatic activity on suspended *L. monocytogenes* cells and against biofilm formation at a concentration of 0.625 mg/mL. These results demonstrate the potential of the produced BLIS as a biopreservative in the food industry.

## 1. Introduction

*Listeria monocytogenes* has a significant impact on food safety and public health, especially in the food industry. These Gram-positive bacteria can survive under various environmental stress conditions, such as temperature, pH, salt concentration and presence of biocides. It can also occur both in food and food chain production, leading to many health and economic losses [1]. Additionally, these microorganisms are able to form biofilm structures, which are resistant to disinfectants (e.g., peracetic acid) and can lead to the persistence of microbial cells and the formation of secondary biofilms on various surfaces [2].

Listeriosis is the infection caused by this pathogen, which can result in serious complications with high hospitalization and mortality rates [3]. Contaminated food is the most important route of transmission of these microorganisms to consumers [4]. The growing trend towards the consumption of minimally processed ready-to-eat foods (RTE) may result in some complications regarding the safety and quality of these products since they can become more susceptible to microbial contamination due to longer shelf-life and the absence of proper food preservatives [5]. In this context, to control *L. monocytogenes* and other *Listeria* species in food production environments, effective monitoring programs and control strategies are essential [6]. The resistance of these pathogens to conventional antibiotics is another attention point that stimulates the search for alternative control methods [7,8].

Among the antibiotic alternatives to control these pathogens are the use of endolysins from bacteriophages, essential oils, plant extracts, antimicrobial peptides and bacteriocins [4,9,10]. Bacteriocins are antimicrobial peptides produced by many bacteria to compete with other bacterial strains. They are extremely diverse and play a significant role in specific ecological niches. In general, bacteriocins exhibit a narrow antimicrobial spectrum, showing antimicrobial activity toward a single species, particularly against species that are phylogenetically related to the producer [11]. Nowadays, bacteriocins are gaining interest as natural food preservatives due to their antimicrobial activities against resistant strains, such as foodborne pathogens, and also considering the growing demand for clean-label products [12]. Before its complete purification and characterization, a substance displaying such antimicrobial activity is usually called a bacteriocin-like inhibitory substance (BLIS) [11].

To produce bacteriocins, complex and expensive culture media such as the De Man, Rogosa and Sharpe (MRS) medium are often used [13]. The need to make production economically competitive and sustainable encouraged the search for alternative culture media since the culture medium can represent up to 30% of the total production cost in commercial fermentations [14]. So, this aspect strongly influences the technical–economic viability of bacteriocin production.

In this way, due to the abundance of fermentable sugars, high availability in the national territory and low price, sugarcane molasses (SCM) represents a promising alternative as a low-cost substrate for biotechnological processes [15]. The sugarcane industry plays a vital role in the Brazilian economy and global food trade. According to an estimate referred to the 2023/24 harvest [16], Brazil is the largest sugarcane producer in the world, with a production of 677.6 million tons of sugarcane. It is estimated that between 40 and 60 kg of molasses are produced per ton of processed sugarcane. 

The use of low-cost carbon sources for biomolecule production is a strategy to reduce manufacturing process costs, as well as to recycle fermentable sugars from agro-industrial waste biomass. Thus, the valorization of agro-industrial residue and by-products through the biotechnological production of high-value-added microbial metabolites such as bacteriocins has shown great potential. Obtaining lactic acid bacteria (LAB) biomass by SCM fermentation has also been explored to simultaneously produce bacteriocins as value-added antimicrobial products [17,18].

Based on this background, this work aimed to evaluate the anti-listerial activity of BLIS produced by *Enterococcus lactis* LBM BT2 cultivated in an alternative culture medium containing SCM as a low-cost carbon source. Our findings showed that the SCM-based medium resulted in a biomass production comparable to that obtained in standard MRS medium but at a lower cost. It was possible to recover and pre-purify the bacteriocin produced by *E. lactis* LBM BT2, which showed activity against planktonic *L. monocytogenes* and inhibited its biofilm-forming ability. These promising results indicate the potential of using such an agro-industrial by-product as a low-cost alternative carbon source in biotechnological applications for the production of biopreservatives.

## 2. Results

### 2.1. Bacterial Identification and Screening of Enterocin-Encoding Genes by PCR

**Figure 1:**
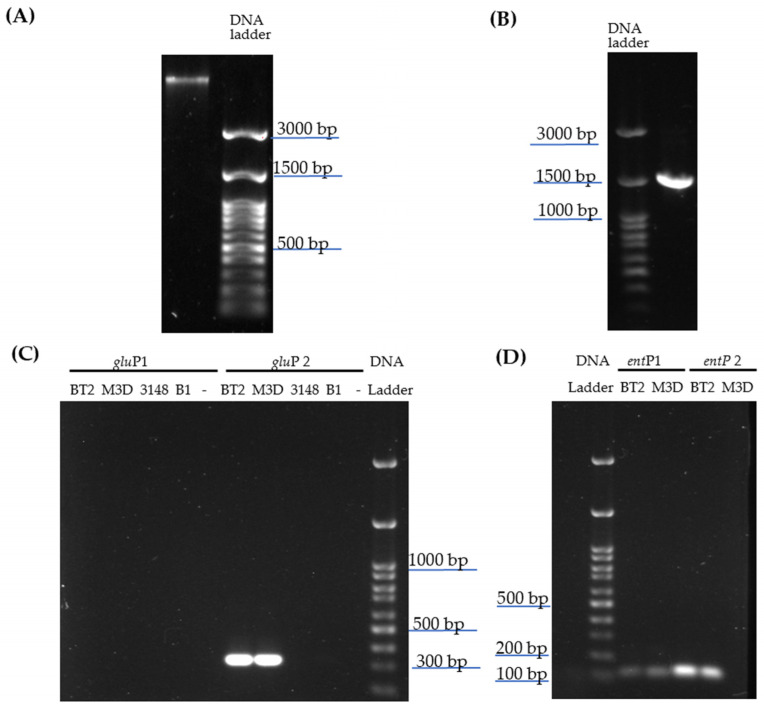
Agarose gel electrophoresis of genomic DNA (DNAg) extraction from strain *E. lactis* LBM BT2 (**A**), 16S rRNA amplification using DNAg from LBM BT2 (**B**), PCR products using *glu*P primer set (*glu*P1 or *glu*P2) (**C**) or entP oligonucleotides (*ent*P1 or *ent*P2) (**D**). As PCR template, it employed DNAg strains from collection of the Microbial Biomolecules Laboratory (LBM), e.g., *E. lactis* LBM BT2 (BT2), *E. lactis* LBM M3D (M3D), *E. faecalis* LBM 3148 or *Lactococcus lactis* LBM B1 (B1). DNA Ladder: 100 bp DNA Ladder RTU (Kasvi, São José dos Pinhais, PR, Brazil).

The genomic DNA (DNAg) was successfully extracted from *E. lactis* LBM BT2 with high purity and integrity, as shown in agarose gel (Figure 1A), as well as by the results of nanodrop (92 ng/µL with 260/280:1.88) and Qibit (90.6 ng/µL) analysis. 

A single band of ~1500 bp was amplified using fD1 and rP1 oligonucleotides and DNAg template (100 ng) (Figure 1B). After PCR product purification, Sanger sequencing was performed with primers covering the V1-V9 16S regions, and the resulting assembly sequence revealed the same similarity values (98.96% E value: 0.0) for many *E. faecium* strains, e.g., HY07 (Accession number: CP032308.1), NK1 (KT278848.1) and HBUAS54298 (MH817747.1), and also for *E. lactis* strains, e.g., E843 (CP082267.1), J-2-A (CP123609.1), SU-B46 (CP 129887.1). For the distinction between *E. faecium* and *E. lactis*, the presence of rhomboid protease gene (*glu*P) variants was checked. An amplification product between 300 and 350 bp was only obtained when the genomes of *E. lactis* LBM BT2 or *E. lactis* LBM M3D and *glu*P2 primers set were used, indicating that the *glu*P variant present in *E. lactis* LBM BT2 actually belongs to *E. lactis* (Figure 1C). No amplification product was observed either when *glu*P1-specific oligonucleotides for *E. faecium* spp. were employed or when DNAg from *E. faecalis* LBM 3148 or *L. lactis* LBM B2 was applied in PCR assay using *glu*P1 or *glu*P2 primer set, thus confirming the test specificity. 

In order to verify enterocin-like genes’ presence in the *E. lactis* LBM BT2 genome, oligonucleotides previously described for amplification of bacteriocin genes present in this species were used. No amplification bands were observed when specific primers for *ent*A and *ent*L50 A and B genes were employed, nevertheless, a PCR product between 100 and 200 bp was amplified from genomes of *E. lactis* LBM BT2 and LBM M3D using *ent*P1 and P2 primers (Figure 1D).

### 2.2. Antibacterial Screening of Bacteriocin-like Inhibitory Substances from E. lactis LBM BT2

The inhibitory effect of BLIS produced by *E. lactis* LBM BT2 was assessed against several pathogenic strains by the agar well diffusion assay. The results listed in Table 1 show that the BLIS had antibacterial activity against all Gram-positive bacteria, including *Listeria* spp. but with exception of *Staphylococcus aureus*. However, no antibacterial effect against all tested Gram-negative bacteria was observed. On the basis of these results, further studies were performed in order to better investigate the BLIS anti-listerial potential.

### 2.3. Enterococcus lactis Growth on Different Substrates

**Figure 2:**
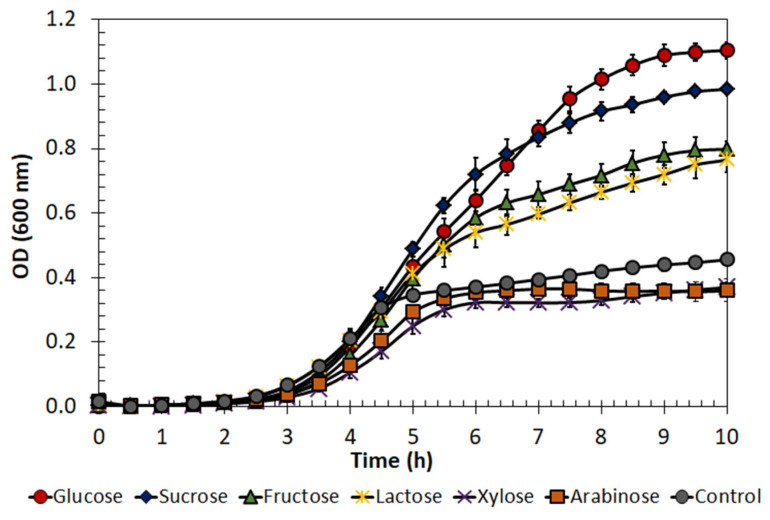
*Enterococcus lactis* LBM BT2 growth (optical density, OD) in MRS-basal medium with different carbon sources. Data are presented as mean ± standard deviation.

The growth of *E. lactis* LBM BT2 on different carbon sources (sucrose, fructose, lactose, xylose and arabinose) was investigated and compared with that on glucose (Figure 2), i.e., the MRS medium standard carbon source. 

The results indicated that the microorganism was able to grow and consume sucrose, fructose and lactose. On the other hand, the pentose sugars seemed not to be assimilated by the microorganism since the optical density (OD) at 600 nm of xylose- and arabinose-based fermented broths was comparable to that of the negative control (without carbon source). It is worth mentioning that MRS medium is a complex medium that also contains other nutrients, such as yeast extract, peptone and meat extract, that can be used for microbial cell growth.

Based on these results and considering the abundance of fermentable sugars in sugarcane molasses (mainly sucrose, but also glucose and fructose), this alternative carbon source was chosen in further tests for *E. lactis* LBM BT2 growth and bacteriocin production.

### 2.4. Molasses Composition

Sugar content (sucrose, glucose and fructose) in sugarcane molasses used in this study was determined through HPLC analyses. Specifically, the contents of total fermentable sugars, sucrose, glucose and fructose were about 69, 44, 13 and 12% (*w*/*w*), respectively.

### 2.5. Effect of Different Molasses Concentration on E. lactis Growth

Microbial growth was then performed in culture media prepared with different concentrations of molasses (2.5, 5.0, 10.0, 15.0, 20.0, 30.0 and 40.0 g/L) in order to know under which conditions the microorganism best adapts itself and/or whether substrate inhibition takes place above a certain concentration. 

For this purpose, the results of the specific growth rate listed in Table 2 show that, although *E. lactis* LBM BT2 was able to grow in all molasses concentrations, substrate inhibition may have occurred at concentrations above 20 g/L. This occurrence is typically observed during batch fermentations in which a high concentration of substrate or product can affect the microbial metabolism for different reasons, including dark color, the presence of suspended particulate matter, too-high osmotic pressure and viscosity. Based on these results and to facilitate the control of operating parameters, such as OD, the molasses concentration of 15 g/L was selected in further BLIS production studies.

### 2.6. Growth and BLIS Production by Enterococcus lactis in Molasses as Carbon Source

After selecting the concentration of molasses that was able to ensure the best growth of *E. lactis* LBM BT2, the suitability of sugarcane molasses for BLIS production was evaluated. For this purpose, 15.0 g/L of molasses was added to the MRS medium as a substitute for glucose, and BLIS production, cell growth and total sugar consumption were evaluated in shake flasks. Since most of the total sugars of molasses are sucrose, a sucrose-based medium was also used as a control.

**Figure 3:**
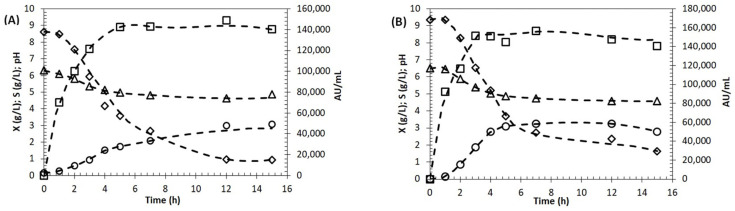
Shake flask cultivation of *E. lactis* LBM BT2 in molasses (**A**) and sucrose-based medium (**B**); cell dry concentration (X (g/L), O); pH variation (Δ); total sugars (S, (g/L), ◊); BLIS activity (AU/mL, □).

The growth curves in Figure 3 show that the highest biomass concentration was similar in both media, reaching approximately 3.0 g/L. Additionally, the exponential growth phase lasted about 3 h in both, and then the specific growth rate decreased slightly, probably due to the pH drop. The BLIS production curve showed that antimicrobial activity reached its maximum value after 5 h (149,000 AU/mL) in cultivation with molasses and after only 3 h in sucrose-based medium (151,000 AU/mL). Regarding the consumption of substrate, it was observed that total sugars were not completely consumed before the cells entered the stationary phase in both cultivations. 

**Figure 4:**
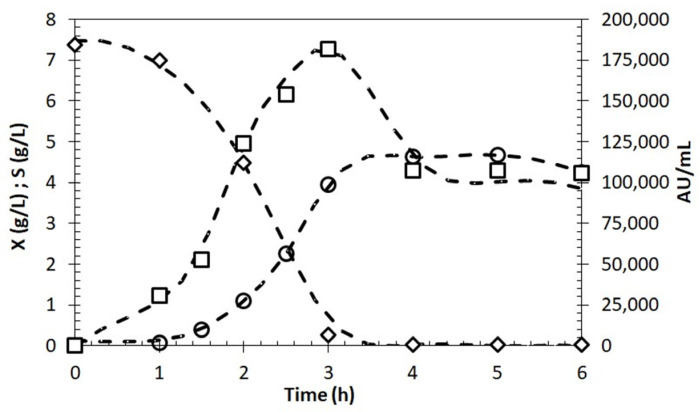
Batch fermentation of *E. lactis* LBM BT2 growth and bacteriocin production in a stirred tank 2 L bioreactor; cell dry concentration (X (g/L), O); total sugars (S (g/L), ◊); BLIS activity (AU/mL, □).

These results, as a whole, indicate that molasses may have a promising potential as an alternative carbon source for bacteriocin production also by other bacteria. However, experiments in shake flasks have some limitations since monitoring and controlling parameters during cultivation are impossible. Thus, an additional experiment was carried out in a stirred tank 2 L bioreactor with pH control to evaluate the effect of scale-up on growth and BLIS production in a molasses-based medium (Figure 4).

The results demonstrated that there was a 53% increase in biomass concentration, which reached a value of 4.6 g/L after only 4 h. This was probably due to the optimal pH maintained during the entire cultivation, which provided optimal growth conditions. In contrast to what was observed in shake-flask experiments, the substrate was completely consumed, and after that, the cell entered the stationary growth phase, probably due to the depletion of carbon sources or other essential nutrients. Regarding BLIS, after achieving its maximum concentration (around 3 h), a decline in production rate followed by a significant decrease in antimicrobial activity was observed, suggesting that the rate of BLIS degradation became higher than that of its production.

### 2.7. Partial Purification and Characterization of BLIS

After selecting the concentration of molasses that was able to ensure the best growth of *E. lactis* LBM BT2, the suitability of sugarcane molasses for BLIS production was evaluated. For this purpose, 15.0 g/L of molasses was added to the MRS medium as a substitute for glucose, and BLIS production, cell growth and total sugar consumption were evaluated in shake flasks. Since most of the total sugars of molasses are sucrose, a sucrose-based medium was also used as a control.

For partial purification of BLIS produced in a shake flask on a molasses-based medium, different amounts of ammonium sulfate (10–60% *w*/*v*) were added to 2 mL aliquots of the cell-free supernatant thermally treated at 80 °C and with pH adjusted to 7.0 (CFS) for protein separation. No relevant difference in the antimicrobial activity was detected using a final saturation from 30 to 60% (*w*/*v*); thus, the lowest concentration of this range (30%) was selected for a subsequent experiment using a larger volume (500 mL). In the next purification step, tangential flow filtration using a membrane cutoff of 2 kDa was performed for salt removal and sample concentration. The retained fraction (>2 kDa) was lyophilized and considered as semi-purified BLIS, while the permeate (<2 kDa) did not exhibit any anti-listerial activity.

**Figure 5:**
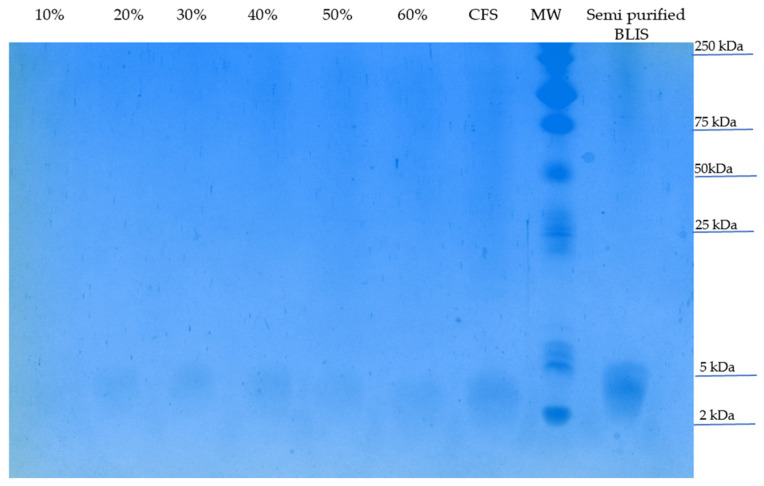
Tricine-SDS-PAGE of partially purified BLIS produced by *E. lactis* LBM BT2 after staining with Coomassie Brilliant Blue G250. Proteins from salted-out (from 10 to 60% *w*/*v*), CFS and semi-purified BLIS samples were separated in 16% of acrylamide gel in the presence of 6 M urea. MW: molecular weight: Precision plus protein dual Xtra Standards (Bio-Rad, Hercules, CA, USA).

Samples of the BLIS-containing fraction were then submitted to Tricine sodium dodecyl sulfate (SDS) polyacrylamide gel electrophoresis (PAGE). Figure 5 shows an evident band between 2 and 5 kDa in all BLIS-containing samples, i.e., CFS, a salted-out sample (from 20 to 60% *w*/*v*) and a semi-purified BLIS sample. Despite the presence of numerous protein bands in the semi-purified BLIS sample, such a level of purification can be considered successful for an initial purification process.

### 2.8. Stability of Semi-Purified BLIS to pH, Temperature and Enzymatic Hydrolysis

The capability of semi-purified BLIS from *E. lactis* LBM BT2 to maintain its own antibacterial activity under different conditions was then evaluated. As shown in Table 3, the antibacterial effect was kept after incubation under different conditions of pH (2.0, 4.0, 8.0 and 10.0) and temperature (60, 80, 100 and 121 °C), which suggests that BLIS may be considered a heat-stable (up to 100 °C) and pH-tolerant substance. On the contrary, this semi-purified product was unstable at 121 °C and after enzymatic treatment. Moreover, the loss of antimicrobial activity detected after treatment with proteases (proteinase K and trypsin) is consistent with the proteinaceous nature of bacteriocins.

### 2.9. Effect of Semi-Purified BLIS on Listeria monocytogenes Growth

**Figure 6:**
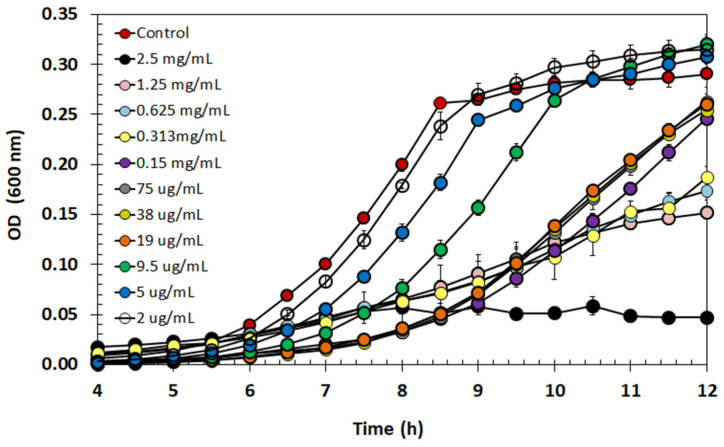
*Listeria monocytogenes* growth (optical density, OD) in BHI medium with different concentrations of the semi-purified BLIS produced by *Enterococcus lactis* LBM BT2. Data are presented as mean ± standard deviation.

The antibacterial activity of semi-purified BLIS was tested in a BHI medium against suspended (planktonic) cells of *L. monocytogenes*. One can see in Figure 6 that there was an inhibiting effect on the growth under almost all tested conditions. Particularly, the pathogen growth was strongly inhibited at the highest concentration of semi-purified BLIS (2.5 mg/mL) compared to the control, but inhibition was gradually reduced at progressively higher dilutions up to approaching the control growth curve. No tested concentration had a bactericide effect, suggesting that semi-purified *E. lactis* BLIS has a bacteriostatic action.

### 2.10. Biofilm Inhibition Activity of Semi-Purified BLIS

**Figure 7:**
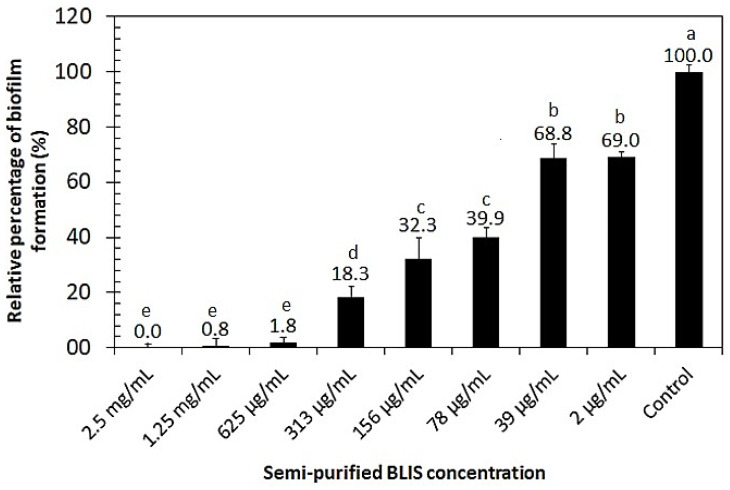
Inhibitory effect of semi-purified BLIS from *Enterococcus lactis* BT2 against *Listeria monocytogenes* biofilm formation. Average values with different superscripts indicate significant differences (*p* < 0.05).

The biofilm inhibition activity of semi-purified BLIS was tested at different concentrations and incubated at 37 °C using the crystal violet assay. As depicted in Figure 7, the capability of *L. monocytogenes* to form biofilm was totally suppressed at the highest concentrations of semi-purified BLIS tested (0.625–2.5 mg/mL). All average values submitted to the Tukey test (*p* < 0.05) showed significant differences compared with the positive control.

## 3. Discussion

### 3.1. Bacterial Identification and BLIS Characterization

Bacteriocins produced by enterococci have stood out due to their abundance and variability, with representatives in all classes of these biomolecules [19,20]. Almeida-Santos et al. [19] reported 19 types of bacteriocins produced by *E. faecalis* and 24 by *E. faecium*. Interestingly, examples of class I, IIa/b/c/d and III of bacteriocins produced by *E. faecalis* have been described in the literature, and the most recurrent species found as class IIa enterocin producer is *E. faecium* (e.g., Enterocin A/P/MC4-1/M, Bacteriocins 43, GM-1, 31, RC714, E 50–52 and T8) [19]. 

A recent taxonomic classification identified *E. lactis* as a novel enterococcus based on the 16S rRNA gene using *E. faecium*-type strain NCTC 7171 as a reference. Nevertheless, several *E. faecium* strains from clade B were not considered, resulting in a misidentification of some isolates from hospital samples as well as in probiotic products [21]. Other identification approaches have been proposed for detection and differentiation between these species since phylogenetic analysis based on their 16S rRNA gene sequences did not form separated clades [22]. Belloso Daza et al. [23] successfully developed a rapid PCR assay using specific primers for rhomboid protease gene variants displaying 100% accuracy validated against well-genotypically and phenotypically characterized isolates. Figure 1 shows a single amplification product containing 300–350 bp only when *glu*P *E. lactis* primers (*glu*P2) were employed, while no amplification bands were detected using *glu*P *E. faecium* primers (*glu*P1). This PCR product size is similar to those previously found in *E. lactis* amplification [23], indicating that the LBM BT2 strain likely belongs to this enterococci species. Unlike the majority of *E. faecium* strains described in the literature, in general, *E. lactis* lineages present greater susceptibility to antibiotics and do not harbor key virulence factors [23].

Tedim et al. [24] developed an enterococcus bacteriocin database using 997 *E. faecium* and *E. lactis* genomes. It was observed that class IIa and IIb were predominant in *E. faecium* genomes with 73.1% of *ent*A gene, 38.7% *bac*AS3 and 38.5% *bac*AS32, whereas for GM-1 (entorocin-P-like), *ent*L50A and *ent*L50B were the only bacteriocins positively associated with *E. lactis* [24]. Thus, specific primers for these bacteriocins more frequent in *E. lactis* were employed in PCR assays. As observed by Kang and Lee [25], a PCR fragment of 100–150 bp was obtained when a P2 primer set was used, indicating that the LBM BT2 strain harbors the structural enterocin P gene. This bacteriocin belongs to class II (small heat-stable nonlantibiotics) and subgroup IIa (pediocin-like bacteriocins) with a theoretical molecular weight of 4.493 kDa. After partial BLIS purification, a peptide band between 2 and 5 kDa in Tricine-SDS-PAGE was observed (Figure 5), as previously found in several enterococci class II bacteriocins partially purified with ammonium sulfate precipitation [26]. 

The results of semi-purified BLIS stability under different pH and temperatures (Table 3) were shown to be similar to those of a BLIS containing enterocin P produced by *E. faecium* P13, with the exception of temperature treatment at 121 °C [27]. Loss of anti-listerial activity of semi-purified BLIS was observed after incubation at this temperature (Table 3), while *E. faecium* P13 BLIS was heat stable at 121 °C. This divergence may have occurred due to the longer incubation period utilized in the present study (15 vs. 30 min). 

It is worth noting that more purification steps should be performed to achieve >95% of pure bacteriocin to finally identify which bacteriocin(s) is(are) produced by the LBM BT2 strain since *E. lactis* spp. genomes harbor an average of 0–7 copies of bacteriocin genes, and some strains have already been described to produce more than one bacteriocin [24].

### 3.2. BLIS Production by Enterococcus lactis LBM BT2 in Molasses-Based Medium

In this work, an alternative low-cost substrate for *E. lactis* LBM BT2 growth and BLIS production was evaluated. At the beginning of this study, the influence of different carbon sources on cell growth was investigated. Sucrose, fructose, lactose, xylose and arabinose were individually used as the sole carbon source in the MRS basal medium and compared with glucose, i.e., the MRS medium carbon source. The results indicated that the microorganism was able to grow and consume all hexoses and sucrose. Although glucose is considered the main carbon source for all heterotrophic microorganisms due to its favorable molecular size, rapid uptake utilization and cell energy conversion, some bacteria have an enzymatic machinery that allows them to use more complex carbohydrates. Sucrose is a cheaper carbon source than glucose and has already been reported for bacteriocin production by LAB [13,28,29]. Since bacteriocins are generally reported as primary metabolites, the increase in cell density would be a good strategy for enhanced bacteriocin production [30].

Bacteriocin production by LAB is usually performed in MRS broth, a complex medium that promotes good growth and enhanced bacteriocin synthesis [13,14]. However, this medium is considered not suitable for large-scale production and industrial applications mainly due to its high cost. In this context, carbon and/or nitrogen-rich agro-industrial by-products have been successfully used to prepare alternative media for bacteriocin production by these bacteria due to cost reduction and sustainability aspects [14]. Thus, in this study, we proposed the use of sugarcane molasses, a by-product of the sugar and ethanol industry, as a low-cost carbon source for BLIS production by *E. lactis* LMB BT2.

Sugarcane molasses is a viscous, dark and sugar-rich by-product resulting from sugar extraction from sugarcane, which contains about 50–60% (*w*/*v*) total sugars (mainly sucrose, glucose and fructose) and traces of amino acids, vitamins and inorganic salts [31,32]. The potential of molasses-based media for bacteriocin production has already been reported by some authors. Gamasheva et al. [13] reported that the use of low-cost media containing molasses and steep corn liquor resulted in the successful production of enterococci bacteriocins with activity against *L. monocytogenes*. In the same way, Mulyani et al. [33] evaluated an enriched medium containing 30% molasses for the production of an anti-listerial bacteriocin by *Pediococcus pentosaceus* 2A2 and highlighted the potential of molasses as a low-cost substrate. According to them, the utilization of molasses as a substrate for microbial cultivation resulted in satisfactory bacteriocin production and contributed to the reduction in the process costs.

Figure 3 shows that cell growth and BLIS production in a molasses-based medium were similar to those in the sucrose-based one. In both cultivations, the bacteriocin production occurred during the cell growth and reached its maximum value at the end of the exponential growth phase. Similar results were reported by other researchers [34,35,36] who also found growth-associated kinetics for BLIS production. In this context, the results indicate that molasses can be a promising alternative carbon source for BLIS production by *E. lactis* LBM BT2. 

Scaling-up of the production of BLIS was performed through batch fermentation in a 2 L bioreactor with pH control on a molasses-based medium. The results showed that biomass concentration increased by 53.3%. Higher cell concentration was reached probably due to pH maintenance at around 6.5, which provided optimal growth conditions. In shake-flask cultivation, the pH decreased to about 4.5, negatively impacting cell growth. Additionally, the specific growth rate in bioreactor cultivation (1.286 h^−1^) was more than twice the one observed in the shake flask (0.563 h^−1^). It is known that the pH is one of the most important parameters influencing the growth of LAB. Yang et al. [34] found that at pH 4.5, the bacteriocinogenic LAB were not able to grow, and the effect of initial pH and temperature strongly influenced cell growth as well as bacteriocin production. 

The production of BLIS in the bioreactor reached a value as high as 181,000 AU/mL at the end of the exponential growth phase, which corresponded to a 26.6% increase compared with shake-flask fermentation. However, after achieving maximum production, a decrease in antimicrobial activity was observed. Similar results were reported by Herranz et al. [35], who studied the enterocin P production by *E. faecium* P13 in batch cultivation with and without pH control. They observed a four-fold increase in cultivations at constant pH 6.0 compared with uncontrolled pH cultivation, followed by an inactivation of antimicrobial activity. According to the authors, the decrease in bacteriocin activity may be explained by its adsorption onto the producing cells, proteolytic inactivation and/or aggregation of monomers, producing less active oligomers.

Some authors agree that the highest bacteriocin quantities are generally obtained at pH and temperature values lower than the optimum ones for biomass production. According to Ünlü et al. [36], bacteriocin production is generally associated with microbial growth; however, improvement in biomass concentration does not guarantee higher bacteriocin production. The apparent contradiction between growth association and non-growth association of bacteriocin production may be due to the adsorption of bacteriocins onto the cell surface and/or proteolytic bacteriocin degradation, both phenomena being related to the pH of the medium [34,35,37].

Thus, the results indicated the potential of sugarcane molasses as a low-cost carbon source for bacteriocin production by *E. lactis* LBM BT2 and highlighted the importance of culture medium composition and monitoring operating parameters, such as pH and temperature, to improve antimicrobial production. Since the optimization of production is an important step in the industrial production of any product, future studies are needed to develop an optimized culture medium in order to support the physiological demands of the strain and increase bacteriocin production.

### 3.3. Anti-listerial Activity of BLIS Produced by Enterococcus lactis LBM BT2

The spectrum of action of BLIS from *E. lactis* LBM BT2 was assessed against different bioindicator bacterial strains. The BLIS antibacterial activity against almost all tested Gram-positive bacteria, with the exception of *S. aureus* CECT239, and no activity against the Gram-negative ones are in line with other studies [38,39]. Several authors have reported that most bacteriocins isolated from Gram-positive bacteria inhibit closely related species to the producer and, usually, do not harbor an inhibition effect over Gram-negative bacteria, whose resistivity could be explained by the outer membrane presence that likely prevents bacteriocin passage through the cytoplasmic membrane [40,41].

When tested at different concentrations against *L. monocytogenes*, the target foodborne pathogen selected in this study, the semi-purified BLIS, showed a growth-inhibition effect. As shown in Figure 6, at a concentration higher than 19 µg/mL, the antimicrobial compound seemed to keep the pathogen in the stationary phase with lower OD in comparison to the control. Although no concentration was able to completely kill the bacterial cells, the highest concentration (2.5 mg/mL) was responsible for an almost complete suppression of *L. monocytogenes* growth. Thus, it was assumed to be the minimum inhibitory concentration of semi-purified BLIS. In this way, these findings suggest that the semi-purified BLIS has a bacteriostatic action, simply preventing the growth of *L. monocytogenes*. 

Bacteriocins are usually reported to have bactericidal/bacteriostatic action and are affected by many factors, including dose, level of purity, indicator/pathogenic and environmental factors [39]. Regarding product purity, some authors stressed that using highly purified bacteriocins is not an economically feasible approach for biopreservatives since it considerably increases the price of products [36,42]. Enterocin P, which was probably the bacteriocin produced by *E. lactis* LBM BT2, is known as an anti-listerial bacteriocin [43,44]. Thus, these findings corroborate the information documented in the literature. 

*L. monocytogenes* is a foodborne pathogen of particular concern, especially in ready-to-eat food products, mainly due to its capability to survive under challenging environmental conditions. In this context, with the increasing consumption of ready-to-eat products and the higher demand for natural foods, biopreservation has become an alternative to traditional chemical methods [36,41,42]. Biopreservation is the term employed to describe the use of antimicrobial compounds derived from microorganisms, such as bacteriocins, in food preservation [45]. According to Todorov et al. [42], the search for new bacteriocins as potentially effective biopreservatives is a growing scientific field due to recent trends toward more natural food products and the increasing demand by consumers for chemical-free and minimal processed food products.

Biofilms are communities of microorganisms embedded in a self-produced matrix, making them resistant to antimicrobial agents [46]. In fact, the formation of *L. monocytogenes* biofilm is one of the most serious disinfection issues in food processing [47]. Therefore, it is important to prevent and eradicate the development of biofilms by this pathogen. In this sense, we demonstrated the capability of semi-purified BLIS against biofilm production by *L. monocytogenes*. Similar results were found in other studies [46,48,49]. 

According to Qiao et al. [50], the prevention of biofilm formation may not involve the direct killing of bacteria or the inhibition of bacterial growth. The authors observed that the bacteriocin BMP32r produced in an *Escherichia coli* expression system reduced the metabolic activity and the number of adherent bacterial cells at the stage of early biofilm formation and maturation. Similarly, the results of the semi-purified BLIS effect on *L. monocytogenes* growth indicated that, despite the growth of suspended cells, no biofilm formation occurred at a concentration higher than 0.3 mg/mL. Therefore, these results point out the anti-listerial capacity of semi-purified BLIS produced by *E. lactis* LBM BT2 not only against planktonic bacteria but also as an inhibitor of biofilm formation. Considering the pH and thermal stability results discussed here, the semi-purified BLIS showed a potential for use as a biopreservative in the food industry, where it is required that antimicrobial products are capable of inhibiting the growth of pathogens and, at the same time, keeping their bioactive activity under different environmental conditions.

## 4. Materials and Methods

### 4.1. Bacteria Strains and Maintenance

The strain *Enterococcus lactis* LBM BT2 used in this work was provided by the collection of the Microbial Biomolecules Laboratory (LBM) at the University of São Paulo. The bioindicator strains *Listeria monocytogenes* CECT 934, *Carnobacterium piscicola* CECT 4020 and *Staphylococcus aureus* CECT239 were acquired from the Spanish Type Culture Collection (CECT). *Escherichia coli* ATCC 25922 and *Pseudomonas aeruginosa* ATCC 27853 were obtained from the American Type Culture Collection (ATCC), and *Listeria innocua* CLIST 2052 was acquired from the Listeria collection (CLIST) of Oswaldo Cruz Institute (FIOCRUZ). *Enterococcus lactis* LBM BT2 was stored in MRS broth (Kasvi, São José dos Pinhais, PR, Brazil), while pathogenic strains were maintained in BHI medium (Kasvi). All bacteria stocks were stored at −80 °C in their respective culture media enriched with 20% glycerol.

### 4.2. Sugarcane Molasses

The sugarcane molasses was obtained from Esther ethanol and sugar plant (Cosmópolis, SP, Brazil) and was stored in a polyethylene container at 4 °C. Fructose, glucose and sucrose contents in molasses were determined by high-performance liquid chromatography (Section 4.10). No additional treatments were carried out before using this substrate in the culture medium.

### 4.3. 16S Ribosomal RNA Identification and Screening of Enterocin-Encoding Genes by PCR

Genomic DNA samples were extracted using PureLink Genomic DNA Mini Kit (Thermo Fisher Scientific, Carlsbad, CA, USA) following Gram-positive bacteria protocol. Nucleic acid integrity was checked in 1% agarose gel and quantified by OD using a NanoDrop 1000 spectrophotometer and by Qubit 2.0 (Thermo Fisher Scientific). A specific set of primers (Table 4) was used to amplify 16S rRNA [49], rhomboid protease [23] and enterocin-like genes previously described in *Enterococcus* spp. [25]. The amplification reaction was carried out according to PCR Supermix (Thermo Fisher Scientific) or GoTaq G2 Green Master (Promega, Madison, WI, USA) instructions. The resulting amplicons of 16S rDNA were purified, sequenced on ABI Prism 3700 DNA sequence (Applied Biosystems, Foster City, CA, USA) and finally compared with the National Center for Biotechnology Information (NCBI) database by using BLASTN. The presence or absence of enterocin-like gene amplicons was verified in agarose gel (1% *w*/*v*) using *E. lactis* LBM M3D and *L. lactis* LBM B1 as positive and negative controls, respectively.

### 4.4. Inoculum and Culture Medium for E. lactis BT2 Cultivation

The kinetics of *E. lactis* growth in MRS basal medium was determined on different carbon sources. The composition of the basal medium was as follows (g/L): peptone, 10; meat extract, 10; yeast extract, 5; K_2_HPO_4_, 2; ammonium citrate, 2; sodium acetate, 5; MgSO_4_·7H_2_O, 0.2; MnSO_4_·2H_2_O, 0.05; and polysorbate 80, 1. After sterilization (121 °C, 15 min) and adding the selected carbon source, the pH of the medium was adjusted to 6.5 ± 0.3 with the addition of NaOH 2 M or HCl 1 M under aseptic conditions. The inoculum was prepared by adding the cryopreserved microorganism at a 2% (*v*/*v*) ratio in a 50 mL Erlenmeyer flask containing 20 mL of MRS-basal medium with 20 g/L sucrose. The incubation time for inoculum was determined as the cells reached the middle of the exponential growth phase, measured through optical density (600 nm) measurements. 

### 4.5. Evaluation of E. lactis LBM BT2 Growth in Different Carbon Sources

*E. lactis* LBM BT2 was cultured in an MRS basal medium and supplemented with 20 g/L of the corresponding carbon source (glucose, sucrose, fructose, lactose, xylose and arabinose). For comparison purposes, a medium with no carbon source was also tested. The inoculum prepared as described in the previous section was added at a 2% (*v*/*v*) ratio. The growth profile was obtained by OD readings at 600 nm in 96-well plates incubated in a microplate reader (BioTek Synergy HTX Multimode Reader, Agilent Technologies, Santa Clara, CA, USA) at 37 °C. Each condition was tested in triplicate.

### 4.6. Impact of Molasses Concentration on E. lactis LBM BT2 Growth

*E. lactis* LBM BT2 growth was evaluated at different concentrations of molasses (2.5, 5.0, 10, 15, 20 and 30 g/L) in an MRS basal medium. Firstly, a 100 g/L molasses solution was prepared and centrifuged after sterilization at 121 °C for 15 min. After appropriate dilution, each molasses solution was separately added to the basal medium. The inoculum was prepared as previously described in Section 4.4 and added at a concentration of 2% (*v*/*v*). The growth profile was obtained as described in the previous section. Each condition was tested in triplicate.

The maximum specific growth rate (*µ*_x_) was estimated as the slope of the straight line resulting from linear fitting of the Neperian logarithm (ln) of cell concentration (*X*) to the cultivation time (*t*) during the exponential growth phase, according to Equation (1):

ln *X* = ln *X*_o_ + *µ*_x_ *t* (1)

where *X*_o_ is the starting biomass concentration.

### 4.7. Kinetics of Growth and Bacteriocin Production in Shake Flasks

Bacteriocin production experiments were carried out in 250 mL Erlenmeyer flasks containing 100 mL of basal medium supplemented with 15 g/L molasses. The inoculum suspension prepared as previously described was added to the flasks at a 5% (*v*/*v*) ratio. A control was also prepared by adding sucrose instead of molasses to the same basal medium. The run was carried out in triplicate by incubation in an orbital shaker at 37 °C and 150 rpm for 15 h. Samples were taken every hour to determine pH, OD, biomass concentration (Section 4.9), sugar consumption (Section 4.10) and antimicrobial activity (Section 4.11). Substrate consumption, cell growth and BLIS production were plotted as functions of time (h), and data were smoothed with a cubic spline function. 

### 4.8. Batch Cultivation in 2 L Bioreactor

Batch cultivation was carried out in a 2 L Biostat B fermenter (Sartorius-Stedim Biotech, Göttingen, Germany) equipped with a pH electrode (EasyFerm VP pH Probe, Hamilton, Reno, NV, USA). The fermenter was sterilized by autoclaving at 121 °C for 20 min. The molasses-based medium (1.5 L) was transferred after pH adjustment into the fermenter under sterile conditions. The inoculum suspension prepared as previously described was added at a 5% (*v*/*v*) ratio. Both temperature and pH were controlled online. In particular, temperature was set at 37 °C, while pH was set at 6.5 ± 0.3 and controlled by automatic addition of NaOH 10 M. Moderate agitation (200 rpm) was performed to ensure homogeneity of the broth. Sampling was performed at appropriate time intervals under sterile conditions from the fermentation vessel to determine pH, OD at 600 nm, biomass concentration, sugar consumption and antimicrobial activity.

### 4.9. Biomass Determination

Cell dry biomass was determined by filtration through membranes with 0.22 μm pore diameter of a known volume of the fermented broth. After that, membranes with cells were dried to constant mass at 105 °C. Cell concentration was determined using a calibration curve of cell dry weight versus OD.

### 4.10. Sugars Analyses

Sucrose, glucose and fructose concentrations in the fermentation broth were separated and detected using a high-performance liquid chromatography (HPLC) (1260 Infinity II LC System, Agilent Technologies, Santa Clara, CA, USA) equipped with refractive index detector operated at 35 °C. The sample volume of 100 µL was injected onto an ion-exclusion column Supelcogel H (9 µm, 30 cm × 7.8 mm) (Sigma-Aldrich, Saint Louis, MO, USA), operated at 30 °C. Components were eluted isocratically with 0.1% phosphoric acid at a flow rate of 1.0 mL/min. Sugars were quantified through calibration curves prepared with standard solutions of glucose, fructose and sucrose at concentrations ranging from 0.5 g/L to 15.0 g/L. 

### 4.11. Antimicrobial Activity Quantification

Antimicrobial activity was quantified using the agar diffusion method with *L. monocytogenes* CECT 934 as a bioindicator. The cell-free supernatant (CFS) was obtained by centrifuging the fermented broth at 5000× *g* and 4 °C for 15 min and adjusting the pH to 6.0–6.5 with 2 M NaOH. Then, the supernatant was heated in a water bath at 80 °C for 10 min to inactivate proteases [52].

In order to prepare the bioindicator inoculum, the culture of *L. monocytogenes* CECT 934 in the exponential growth phase was diluted to an OD (600 nm) of 0.2, which corresponded to approximately 1–5 × 10^8^ CFU/mL. The total count of viable cells was undertaken with the spread plate technique. After OD adjustment, 150 μL of the standardized suspension was added to 15 mL of MRS soft agar (0.8%, *w*/*v*) previously poured into Petri dishes. A serial two-fold dilution of each sample was prepared in PBS buffer at pH 7.0, and then 60 μL of the antimicrobial sample was added to 6 mm wells, previously cut in the agar, by using the back of a sterile 200 μL tip. The plates were incubated at 37 °C for 16 to 18 h. Afterward, the antimicrobial activity (*A*_a_) (AU/mL) of CFS was calculated according to [52], with some modification (Equation (2)):

(2) $$A_{a}=\frac{\pi r^{2}}{v\;}D$$

where *r* is the radius (mm) of the inhibition zone surrounding the wells measured with a caliper, *v* is the sample volume (mL) added to the wells, and *D* is the last dilution in which the inhibition halo could be observed.

### 4.12. Spectrum of Action and Antimicrobial Activity of Cell-Free Supernatant

The antimicrobial activity of CSF prepared as described above was tested by the disc diffusion assay, as described by the Clinical and Laboratory Standards Institute [53], with some modifications. Firstly, the bioindicator strains (Section 4.1) were reactivated overnight in the BHI medium. Then, bacteria were cultivated in fresh BHI broth, and their growth was accompanied until cells reached a turbidity equivalent to 0.5 McFarland standard (corresponding to 10^8^ CFU/mL). The standardized inoculum was inoculated onto the surface of a Mueller–Hinton agar plate using a sterile cotton swab. Before adding the discs, inoculated plates were held at room temperature under aseptic conditions for 10 min to allow evaporation/adsorption of free surface liquid.

Sterile paper discs (6 mm) previously dipped into CFS were placed onto the surface of inoculated agar plates. The corresponding antibiotic disc was used as a positive control for each strain. The plates were incubated at 37 °C for 24 h. After incubation, the diameter of the zone of growth inhibition was measured with a caliper [54]. Each condition was tested in duplicate.

### 4.13. Partial Purification of BLIS

BLIS obtained from *E. lactis* LBM BT2 culture in a molasses-based medium was incubated at 10 °C under agitation (100 rpm) for 2 h in the presence of ammonium sulfate (Labsynth, Diadema, SP, Brazil) at a ratio ranging from 10 to 60% (*w*/*v*). Samples were centrifuged at 13,000× *g* and 4 °C for 30 min, and the resulting pellets were resuspended in PBS (pH 7.0) up to the original BLIS-sample volume. Antimicrobial activity was assessed as described in the previous section, and the lowest concentration of ammonium sulfate, ensuring the highest anti-listerial activity, was selected for the next purification step. For sample desalting and concentration, tangential flow filtration with a molecular weight cutoff of 2 kDa (Sartorius AG, Göttingen, Germany) was employed at a flow rate of 12 mL/min until the retentate sample reached 10% of the initial volume. The antimicrobial activity was assayed in the resulting samples of both retentate and permeate. The retentate sample (>2 kDa) was lyophilized (semi-purified BLIS) and then used in stability and antimicrobial tests.

Proteins/peptides bands were analyzed by Tricine-SDS-PAGE using 16% acrylamide and 1% bisacrylamide in the presence of 6 M urea [55]. For sample preparation, 50 µL of loading buffer [12% SDS (*w*/*v*), 6% mercaptoethanol (*v*/*v*), 30% glycerol (*w*/*v*), 0.05% Coomassie blue G-250 (*w*/*v*), 150 mM Tris/HCl (pH 7.0)] and 150 µL of test samples were incubated for 10 min at 37 °C. Then, 3 μL of each sample was loaded in the respective sample wells. Reagents used for casting gel, sample and gel buffers were acquired from Sigma-Aldrich (Saint Louis, MO, USA). Electrophoresis was run using an initial voltage of 30 V until samples entered the stacking gel and then 90 V up to the end of the run. Finally, gels were incubated in fixing Coomassie staining and destaining solutions for protein visualization, as described by Schägger [55].

### 4.14. Stability of Semi-Purified BLIS to pH, Temperature and Enzymatic Hydrolysis

The susceptibility of semi-purified BLIS to different proteases, pH values and temperatures was evaluated according to Peng et al. [56] with minor modifications. Briefly, for the pH tolerance assay, the pH of samples was adjusted to 2, 4, 8 and 10 using 2 M NaOH or 2 M HCl. Then, after 2 h incubation at room temperature, the pH was adjusted back to 6.5 before the analysis of antimicrobial activity. To analyze the thermal stability of BLIS, samples were incubated at 60, 80 and 100 °C for 30 min and at 121 °C by autoclaving for the same time.

The effect of proteases on semi-purified BLIS was assessed by incubating the samples at 37 °C with trypsin or proteinase K (Sigma-Aldrich, St. Louis, MO, USA) at a final concentration of 3 mg/mL for 2 h. Then, the samples were placed in a water bath at 100 °C for 5 min for enzyme inactivation. For all stability assays, the semi-purified BLIS was suspended in PBS (pH 7.0), and untreated samples were used as positive controls. The antimicrobial activity was analyzed by the diffusion agar method against *L. monocytogenes* CECT 934 (Section 4.11).

### 4.15. Minimum Inhibitory Concentration of Semi-Purified BLIS

The antimicrobial effect of semi-purified BLIS was also evaluated at different concentrations against *L. monocytogenes*. For this purpose, solutions were prepared by two-fold dilutions of semi-purified BLIS with PBS (pH 7). To prepare the inoculum of the pathogen, it was grown in BHI broth at 37 °C until reaching an OD (600 nm) of 0.2. Then, cells were 100 times diluted in order to reach a concentration of 10^6^ CFU/mL. The standardized inoculum was added to each semi-purified BLIS dilution in a proportion of 1:1 and transferred to a 96-well sterile microplate. PBS buffer without antimicrobial compound was used as a positive control. The pathogen growth profile was obtained by OD readings at 600 nm in 96-well plates incubated in a microplate reader (BioTek Synergy HTX Multimode Reader, Agilent Technologies, Santa Clara, CA, USA) at 37 °C. The minimum inhibitory concentration (MIC) value was determined as the lowest concentration of semi-purified BLIS that almost suppressed the growth of *L. monocytogenes*. For each dilution, the experiment was performed in duplicate with three replicate wells per microplate.

### 4.16. Inhibitory Effect of Semi-Purified BLIS on Biofilm Formation by Listeria monocytogenes

Biofilm quantification was performed with a 96-well microtiter plate [45]. Briefly, an overnight-grown inoculum of *L. monocytogenes* was diluted in BHI 2× diluted medium and adjusted to OD at 600 nm of 0.1. An amount of 100 μL of the inoculum was transferred to a 96-well polystyrene microplate with the addition of 100 μL of semi-purified BLIS in each well. The semi-purified BLIS was two-fold serially diluted in PBS (pH 7.0) before being added to the wells. A sterile medium was used as a negative control, and only bacterial suspension as a positive control. Plates were incubated at 37 °C for 24 h.

The inhibiting activity of semi-purified BLIS on biofilm development was determined based on biofilm biomass (crystal violet staining). After incubation for 24 h, the culture medium was discarded, and the wells were washed with sterile NaCl (0.85%) three times to remove the unattached cells. Cells were fixed by the addition of 200 μL of methanol for 15 min and completely air-dried at 55 °C. Then, 0.2% (*w*/*v*) crystal violet solution was added, and after 15 min, the plates were washed with NaCl (0.85%). After drying, 200 μL of 96% (*v*/*v*) ethanol was added for 20 min to solubilize the crystal violet. OD was measured at 595 nm on the microplate reader described above. For each dilution, the experiment was performed in duplicate with three replicate wells per microplate.

### 4.17. Statistical Analysis

All experiments were performed in two or three replicates according to circumstances, and the results were presented as mean ± standard deviation (SD). Statistical analysis was performed using a one-way analysis of variance (ANOVA), and the mean values were compared using Tukey’s test using the Minitab 19 software (Minitab, LLC., State College, PA, USA). The significance level of *p* < 0.05 was considered in all experiments.

## 5. Conclusions

In this study, sugarcane molasses was found to be an interesting, cheap carbon source for the production of an anti-listerial BLIS by *E. lactis* LBM BT2. This is the first report, as far as we are aware, on bacteriocin production by this species using an agroindustrial by-product. The stability tests showed that the semi-purified BLIS maintained its antimicrobial activity in wide pH (2.0–10.0) and temperature (60–100 °C) ranges. These results are promising since food manufacturing usually involves high temperatures (e.g., pasteurization) and different pH conditions. The anti-listerial activity showed that the semi-purified BLIS had a bacteriostatic effect against planktonic cells of *L. monocytogenes* (above 19 µg/mL) and inhibited biofilm formation by the same species at concentrations higher than 625 µg/mL. Since biofilm formation by *L. monocytogenes* can lead to critical situations in the food industry, these results indicated the potential of this BLIS as an alternative, natural anti-listerial antimicrobial. However, future studies are needed to completely purify and characterize it. Moreover, with the growing demand for clean-label products, the study of novel biopreservatives, taking into account food safety agencies’ requirements, requires a great effort from different areas, e.g., genetics, biochemistry and bioprocessing. A deep dive into the whole sequencing of the *E. lactis* LBM BT2 genome would provide important insights regarding genes related to by-product consumption to better explore all potentiality of this bacteriocin-producing strain in terms of circular economy practices. Last but not least, a more detailed understanding of the mode of action of bacteriocins and their possible effects on the host intestinal microbiota when added to foods is also of fundamental importance to enable their use in the food industry as biopreservatives.

## Figures and Tables

**Table 1:** Antibacterial activity of bacteriocin-like inhibitory substance from *Enterococcus lactis* LBM BT2 against Gram-positive and Gram-negative bacteria. Data are presented as mean ± standard deviation.

Pathogenic Strain	Diameter of Inhibition Halo (mm)
*Listeria monocytogenes* CET93	21.05 ± 0.88
*Listeria innocua* CLIST 2052	11.51 ± 0.68
*Carnobacterium piscicola* CECT 4020	11.75 ± 0.91
*Staphylococcus aureus* CECT239	0
*Escherichia coli* ATCC 25922	0
*Salmonella enterica* subsp. *enterica serovar* Typhimurium	0
*Pseudomonas aeruginosa* ATCC 27853	0

**Table 2:** Specific growth rate (*µ*_x_) of *Enterococcus lactis* LBM BT2 in cultivations performed at different molasses concentrations. Average values with different superscripts indicate significant differences (*p* < 0.05).

Molasses Concentration (% *w*/*v*)	*µ*_x_ (h^−1^)
2.5	1.077 ± 0.07 ^a^
5.0	1.070 ± 0.02 ^a^
10.0	1.123 ± 0.02 ^a^
15.0	1.110 ± 0.01 ^a^
20.0	1.101 ± 0.07 ^a^
30.0	0.936 ± 0.02 ^b^
40.0	0.738 ± 0.05 ^c^

**Table 3:** Effect of pH, temperature and enzyme treatments on the stability of semi-purified BLIS. The antibacterial activity, after treatments, was evaluated against *Listeria monocytogenes*. Sign + indicates that the antibacterial activity was maintained under this condition, while the sign—is indicative of the absence of any antibacterial activity.

Treatments		Antibacterial Activity
pH	2.0	+
	4.0	+
	8.0	+
	10.0	+
Temperature	60 °C for 30 min	+
	80 °C for 30 min	+
	100 °C for 30 min	+
	121 °C for 30 min	−
Enzyme	Proteinase K	−
	Trypsin	−

**Table 4:** Primers and conditions used in the polymerase chain reaction (PCR).

Primer Name	Sequence 5′-3′	Target/Product Size	Annealing Temperature	References
*ent*A-For	AAATATTATGGAGTGTAT	Structural enterocin A gene/126 bp	56 °C	[26]
*ent*A-Rev	GCACTTCCCTGGAATTGCTC			
*ent*P1-For	TATGGTAATGGTGTTTATTGTAAT	Structural enterocin P gene/~130 bp		
*ent*P1-Rev	ATGTCCCATACCTGCCAAAC			
*ent* L501-For	STGGGAGAATCGCAAAATTAG	Structural enterocin L50A or L50B genes/130 bp		
*ent* L501-Rev	ATTGCCCATCCTTCTCCAAT			
*ent*P2-For	GCTACGCGTTCATATGGTAATGGTG	Structural enterocin P gene/132 bp	55 °C	[27]
*ent*P2-Rev	ATGTCCCATACCTGCCAAACCAGAAGC			
*ent* L502-For	GATTGGAGGAGTTATATTATGGG	Structural enterocin L50A or L50B genes/556 bp		
*ent* L502-Rev	CAAATTATAAAGAAATAATTACCTATCATTAAC			
fD1	AGAGTTTGATCCTGGCTCAG	16S rRNA gene/ ~1500 bp	60 °C	[51]
rP1	ACGGTTACCTTGTTACGACTT			
*glu*P1-For	GCGTGCATGGTTAAGACGAC	Rhomboid protease gene from *E. faecalis*/427 bp	61 °C	
*glu*P1-Rev	CTGCTGGATCGCTGGGTTAT			
*glu*P2-For	TACGGTCACTGGCGGTTTTT	Rhomboid protease gene from *E. lactis*/324 bp	58 °C	[23]
*glu*P2-Rev	TGTCTGCTGTTTCGGTAGCC			

## Data Availability

Data are contained within the article.
